# Lack of association between a functional polymorphism (rs1800796) in the interleukin-6 gene promoter and lung cancer

**DOI:** 10.1186/1746-1596-9-134

**Published:** 2014-07-01

**Authors:** Weihua Wang, Jie Chen, Feng Zhao, Burong Zhang, Hongsheng Yu

**Affiliations:** 1 The Affiliated Hospital of Medical College of Ningbo University, Ningbo 315020 Zhejiang, China

**Keywords:** Interleukin-6, Lung cancer, Polymorphism, Meta-analysis

## Abstract

**Background:**

A number of studies have examined the association between interleukin-6 (*IL-6*) rs1800796 polymorphism and risk of lung cancer but revealed inconsistent results. The aim of this study was to clarify the association between *IL-6* rs1800796 polymorphism and risk of lung cancer.

**Methods:**

Literature databases including PubMed, Embase and CNKI were searched up to January 2014. The pooled odds ratios (ORs) with 95% confidence intervals (CIs) under co-dominant model, dominant model and recessive model were estimated using random-effects model.

**Results:**

A total of seven studies, including 2691 lung cancer cases and 3067 controls, were included in the meta-analysis. The results suggested that *IL-6* rs1800796 polymorphism was not associated with risk of lung cancer under homogeneous co-dominant model (OR = 1.06, 95%CI = 0.73-1.54), heterogeneous co-dominant model (OR = 1.24, 95%CI = 0.96-1.60), dominant model (OR = 1.23, 95%CI = 0.95-1.58) and recessive model (OR = 0.96, 95%CI = 0.70-1.32). The association was still not significant in either never-smokers (OR = 1.19, 95%CI = 0.95-1.48) or ever-smokers (OR = 1.73, 95%CI = 0.89-3.36).

**Conclusion:**

The present meta-analysis suggested that there was no association between *IL-6* rs1800796 polymorphism and lung cancer, which was independent of smoking status.

**Virtual Slides:**

The virtual slide(s) for this article can be found here: http://www.diagnosticpathology.diagnomx.eu/vs/1060061508127855

## Background

Lung cancer has become a major public health issue worldwide, which accounts for 13% of the total cancer cases and 18% of total deaths in 2008 [1]. To date, the potential mechanism of lung carcinogenesis is not clear. Although it is established that cigarette smoking is one of the most important risk factors causing lung cancer, only 10–15% of heavy tobacco smokers ultimately develop lung cancer [2]. This evidence suggests that genetic factors may play an important role in the development of lung cancer.

Several studies suggested that chronic inflammation predisposes individuals to different types of cancer, including lung cancer. Interleukin-6 (IL-6) is a major cytokine that is known to affect immune response, which is expressed in tumor-infiltrating cells. IL-6 plays a key role in cell survival, proliferation and apoptosis [3]. Three functional polymorphisms (rs1800795 [-174G > C], rs1800796 [-572C > G or -634C > G] and rs10499563 [-6331 T > C]) associated with *IL-6* transcription activity have been identified in the *IL-6* promoter region. To date, a great number of studies have investigated the association between *IL-6* rs1800795 polymorphism and lung cancer, and two meta-analyses [4,5] demonstrated that *IL-6* rs1800795 polymorphism was not associated with risk of lung cancer. In addition, some studies suggested that *IL-6* rs1800796 might be associated with risk of lung cancer; however, the results have been inconsistent [6-11]. To our knowledge, no meta-analysis has been performed to address this topic.

Therefore, in the present study, we aimed to perform a meta-analysis to clarify the association between *IL-6* rs1800796 polymorphism and risk of lung cancer.

## Methods

### Literature and search strategy

PubMed, EMBASE, and CNKI databases were searched for eligible publications. We used the following key words to identify the potential studies: (Interleukin-6 OR IL-6) and (variant OR variation OR polymorphism OR genotype) and lung cancer. Publication language was restricted to English or Chinese. We also hand-searched the reference lists of retrieved article. If duplicative publications existed, only the study with the large sample size was included. The last literature search was on January 1, 2014.

### Inclusion criteria and data extraction

The inclusion criteria for the studies included: (1) evaluated the association of *IL-6* rs1800796 polymorphism with lung cancer; (2) used case–control or cohort design; and (3) provided sufficient data for calculation of odds ratio (OR) with 95% confidence interval (CI). The extracted information from each study included: (1) name of the first author; (2) year of publication; (3) country of origin; (4) sample sizes of cases and controls; (5) genotype distributions of cases and controls; and (6) whether the variant was in Hardy Weinberg Equilibrium (HWE) in controls. Two authors (Weihua Wang and Jie Chen) independently searched articles and extracted the data. The third person was asked if they have different opinions.

### Statistical analysis

The association between *IL-6* rs1800796 polymorphism and risk of lung cancer was estimated by calculation of pooled OR and 95%CI under a co-dominant, a dominant, and a recessive model, respectively. Z test was used to determine the significance of pooled OR (*p* < 0.05 was considered statistically significant). The between-study heterogeneity was evaluated by Q test and and *I*^2^ statistic [12]. A random- [13] or fixed- [14] effects model was performed to calculate pooled OR in the presence (*p* ≤ 0.10) or absence (*p* > 0.10) of heterogeneity, respectively. Subgroup analysis by whether the subjects smoke was performed. Begg’s test [15] was performed to assess publication bias (*p* < 0.05 was considered statistically significant). STATA version 11 (StataCorp LP, College Station, TX, USA) was applied to perform data analysis.

## Results

### Characteristics of the studies

**Figure 1:**
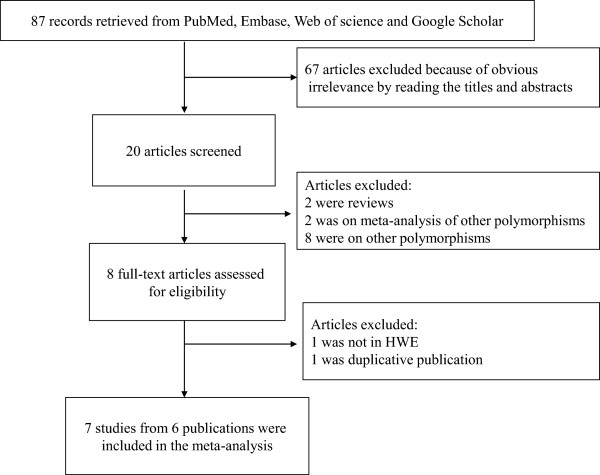
Flowchart of study selection.

After literature search, a total of 87 publications were identified. 67 articles were excluded because of obvious irrelevance after reading abstract or title. In addition, two reviews, two articles on meta-analysis of other polymorphisms and eight articles on other polymorphisms were excluded. Then, one article not in HWE [16] and one duplicated publication [17] were further excluded. At last, a total of seven studies from six publications were included in the meta-analysis (Figure 1). Of seven studies, five were conducted in China, one was conducted in Singapore, and one was conducted in Japan. All study populations were East Asians. The polymorphism in all the included studies was in HWE in controls (all *P* > 0.05). The characteristics of all the included studies are listed in Table 1.

**Table 1:** **Characteristics of the included studies of the association between *IL-6 *** **rs1800796 variant and lung cancer**

**Study**	**Country**	**Ethnicity**	**Sample size**		**Genotype frequency in cases**			**Genotype frequency in controls**			***P ***_**HWE**_
			**Cases**	**Controls**	**GG**	**GC**	**CC**	**GG**	**GC**	**CC**	
Su, 2010 [6]	China	East Asian	363	370	193	156	14	233	117	20	0.30
Lim, 2011 [7]	Singapore	East Asian	298	718	163	123	12	449	231	38	0.25
Bai, 2013 [8]	China	East Asian	193	210	86	89	18	125	69	16	0.15
Chen, 2013 (discovery group) [9]	China	East Asian	622	614	349	229	44	309	252	53	0.87
Chen, 2013 (validation group) [9]	China	East Asian	615	638	333	245	37	321	263	54	0.99
Liang, 2013 [10]	China	East Asian	138	138	100	29	9	105	30	3	0.62
Kiyohara, 2014 [11]	Japan	East Asian	462	379	259	175	28	250	116	13	0.92

*P*_HWE_, *p* value for Hardy-Weinberg Equilibrium test in controls.

### Meta-analysis results

**Figure 2:**
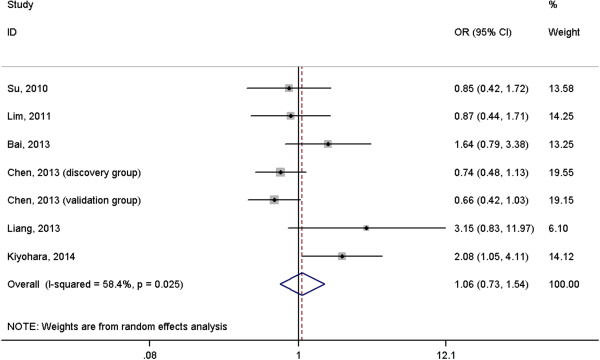
**Forest plot of the association between *IL-6 *** **rs1800796 variant and lung cancer under homogeneous co-dominant model (CC vs. GG).**

**Figure 3:**
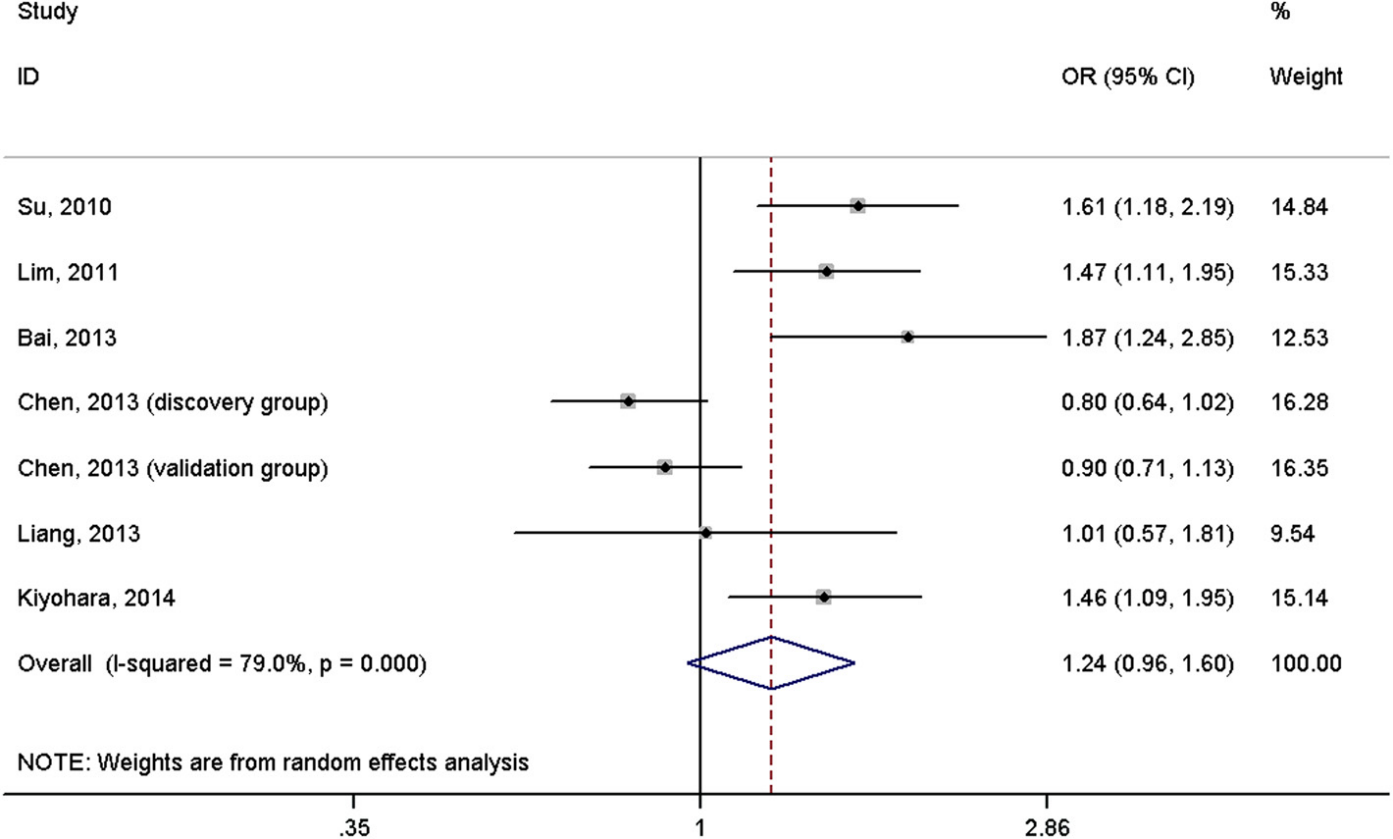
**Forest plot of the association between *IL-6 *** **rs1800796 variant and lung cancer under heterogeneous co-dominant model (GC vs. GG).**

**Figure 4:**
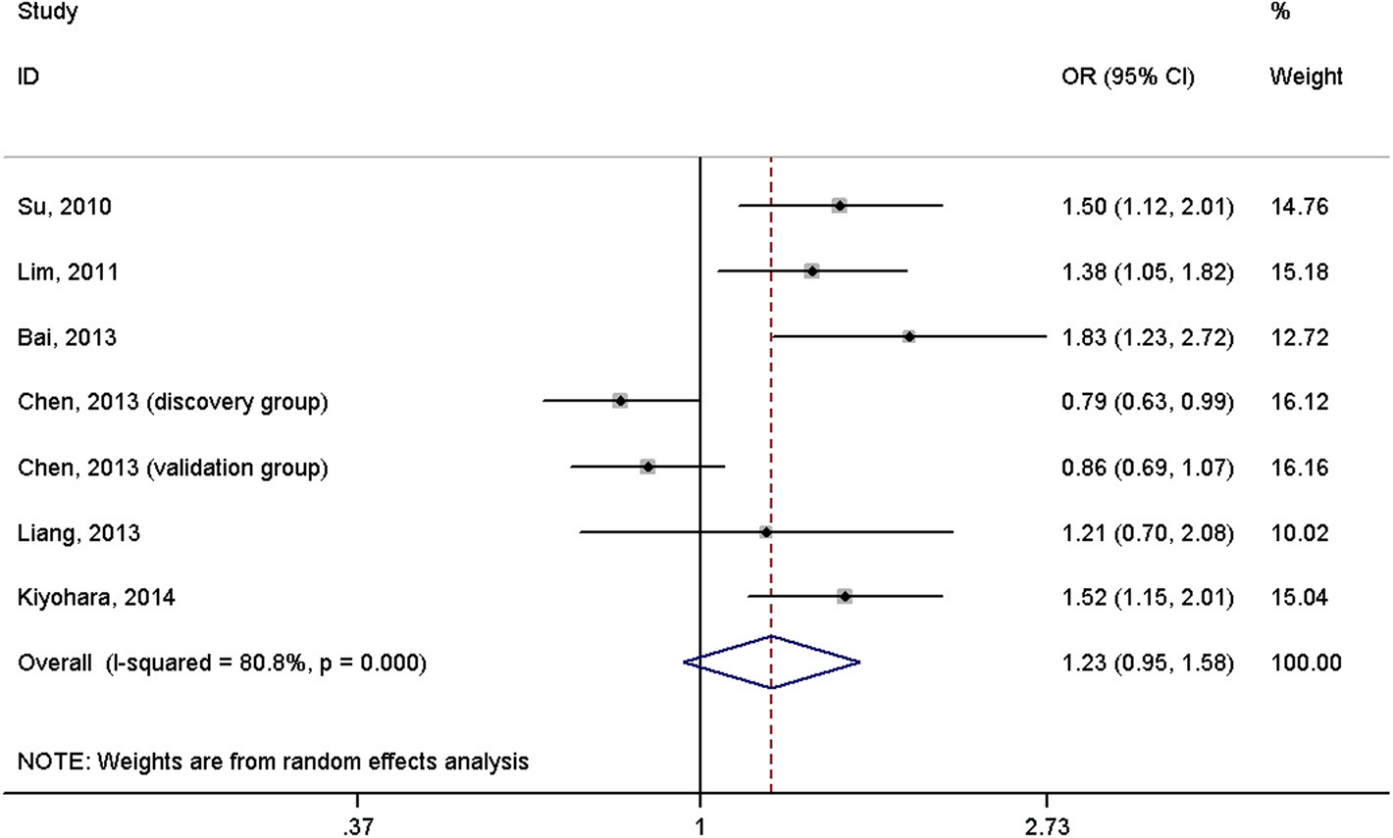
**Forest plot of the association between *IL-6 *** **rs1800796 variant and lung cancer under dominant model (CC + GC vs. GG).**

**Figure 5:**
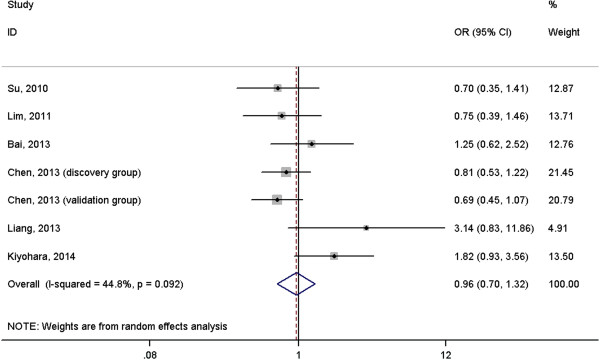
**Forest plot of the association between *IL-6 *** **rs1800796 variant and lung cancer under recessive model (CC vs. GG + GC).**

**Figure 6:**
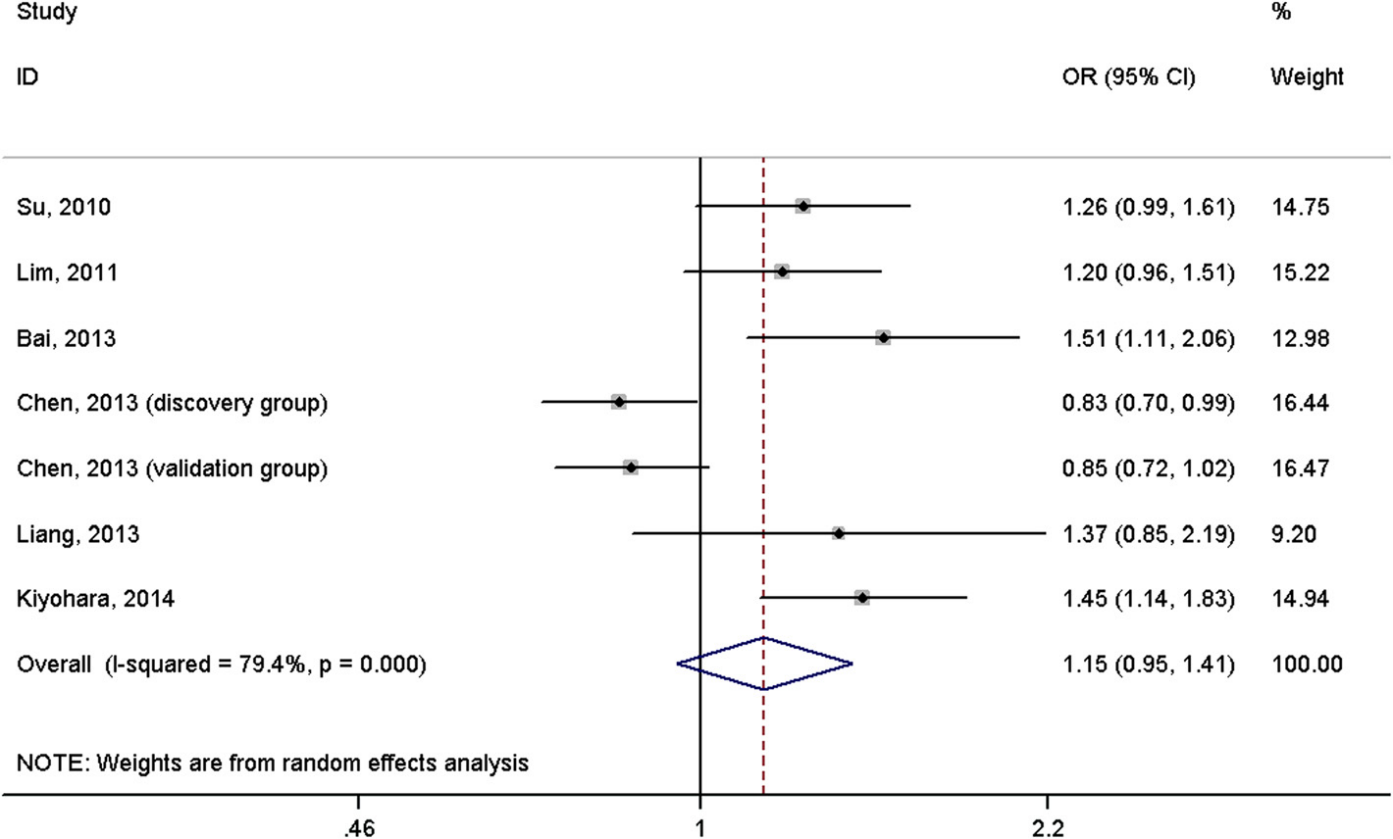
**Forest plot of the association between *IL-6 *** **rs1800796 variant and lung cancer under allelic model (C vs. G).**

A total of 2691 cases with lung cancer and 3067 normal controls were included in the meta-analysis. The results suggested that *IL-6* rs1800796 polymorphism was not associated with risk of lung cancer under homogeneous co-dominant model (OR = 1.06, 95%CI = 0.73-1.54, Figure 2), heterogeneous co-dominant model (OR = 1.24, 95%CI = 0.96-1.60, Figure 3), dominant model (OR = 1.23, 95%CI = 0.95-1.58, Figure 4), recessive model (OR = 0.96, 95%CI = 0.70-1.32, Figure 5) and allelic model (OR = 1.15, 95%CI = 0.95-1.41, Figure 6). Besides using raw genotype data, we also pooled the results adjusted for most common confounding factors under co-dominant model. The non-significant association did not change (homogeneous co-dominant model: OR = 0.94, 95%CI = 0.78-1.14; heterogeneous co-dominant model: OR = 1.21, 95%CI = 0.92-1.59).

Cigarette smoking is a most important risk factor for lung cancer, and it may modify the association between *IL-6* rs1800796 polymorphism and risk of lung cancer, the subgroup analysis stratified by smoking status (no vs. yes) was performed. However, the association was still not significant in either never-smokers (OR = 1.19, 95%CI = 0.95-1.48, Additional file 1: Figure S1) or ever-smokers (OR = 1.73, 95%CI = 0.89-3.36, Additional file 2: Figure S2).

Since there was significant between-study heterogeneity for association between *IL-6* rs1800796 polymorphism and risk of lung cancer under all genetic models, we performed a meta-regression analysis to explore source of heterogeneity. We introduced variables including publication year, genotype methods, sample size in cases and controls. However, these variables cannot explain the source of heterogeneity.

### Potential publication bias

No publication bias was detected for association between *IL-6* rs1800796 polymorphism and risk of lung cancer under all genetic models using the Begg’s test (*P* = 0.133 for homogeneous co-dominant model; *P* = 0.230 for heterogeneous co-dominant model; *P* = 0.230 for dominant model; *P* = 0.230 for recessive model; *P* = 0.368 for allelic model).

## Discussion

The present meta-analysis demonstrated that *IL-6* rs1800796 polymorphism was not associated with risk of lung cancer under all genetic models. In the subgroup analysis, the non-significant association remained in either never-smokers or smokers.

A previous meta-analysis reported that there was no significant association between IL-6 level and lung cancer risk [18]. However, higher level of IL-6 is suggested to be associated with risk of coronary heart disease [19] and type 2 diabetes [20]. In regards to the functional polymorphism rs1800796 in the *IL-6* gene, it was only found to be associated with risk of type 2 diabetes [21], but not with coronary heart disease [22].

It is consistent that rs1800795 polymorphism was not associated nearly all types of cancers, including colorectal [23], prostate [24], gastric [25] and breast cancers [26]. But rs1800796 polymorphism was found to be associated with prostate [24] cancer but not with gastric cancer [25]. Based on 2691 cases with lung cancer and 3067 normal controls, we did find significant association between rs1800796 polymorphism and lung cancer.

Heterogeneity may potentially affect the interpretation of the results. Heterogeneity may be attributed to the potential confounding resulted from publication time, sample sizes, measurement errors, or the interaction with other risk factors. In our study, there was significant between-study heterogeneity for association between *IL-6* rs1800796 polymorphism and risk of lung cancer under all genetic models. However, meta-regression analysis in consideration of the potential confounders did not address the heterogeneity.

There are several limitations in the present study. First, there might be effects of gene–gene and gene–environment interactions [27-29] but we can not address this because the individual studies did not provide the related data. Second, the sample size in the subgroups was limited and the results should be interpreted with caution. Third, we only assessed one polymorphism in the *IL-6* gene, therefore, we can not rule out the possibility that other polymorphisms or haplotypes in this gene might be implicated in the development of lung cancer. Fourth, all the six papers we selected were from East Asia. Thus, the conclusion should not be generalized to other ethnic populations.

## Conclusions

In conclusions, the results of our meta-analysis indicated that there was no significant association between *IL-6* rs1800796 polymorphism and risk of lung cancer, and the non-significant association was independent of whether the individuals smoked cigarettes.

## Competing interests

The authors declare that they have no competing interests.

## Authors’ contributions

WW and JC designed the study and drafted the manuscript. JC and FZ searched the databases and extracted the data. BZ and HY performed the statistical analysis. All authors read, revised and approved the final manuscript.

## Supplementary Material

Additional file 1 **Forest plot of the association between *IL-6 *** **rs1800796 variant and lung cancer in never-smokers with adjustment for potential confounders (dominant model: CC+GC vs. GG).** Click here for file

Additional file 2 **Forest plot of the association between *IL-6 *** **rs1800796 variant and lung cancer in smokers with adjustment for potential confounders (dominant model: CC+GC vs. GG).** Click here for file
