# Supplementary material for: Lack of association between a functional polymorphism (rs1800796) in the interleukin-6 gene promoter and lung cancer
Source: Diagn Pathol. 2014 Jul 1;9:134. doi: 10.1186/1746-1596-9-134 (PMC4100037; doi:10.1186/1746-1596-9-134)
Supplement: Additional file 2 — Forest plot of the association between IL-6 rs1800796 variant and lung cancer in smokers with adjustment for potential confounders (dominant model: CC+GC vs. GG). [file 1746-1596-9-134-S2.doc]

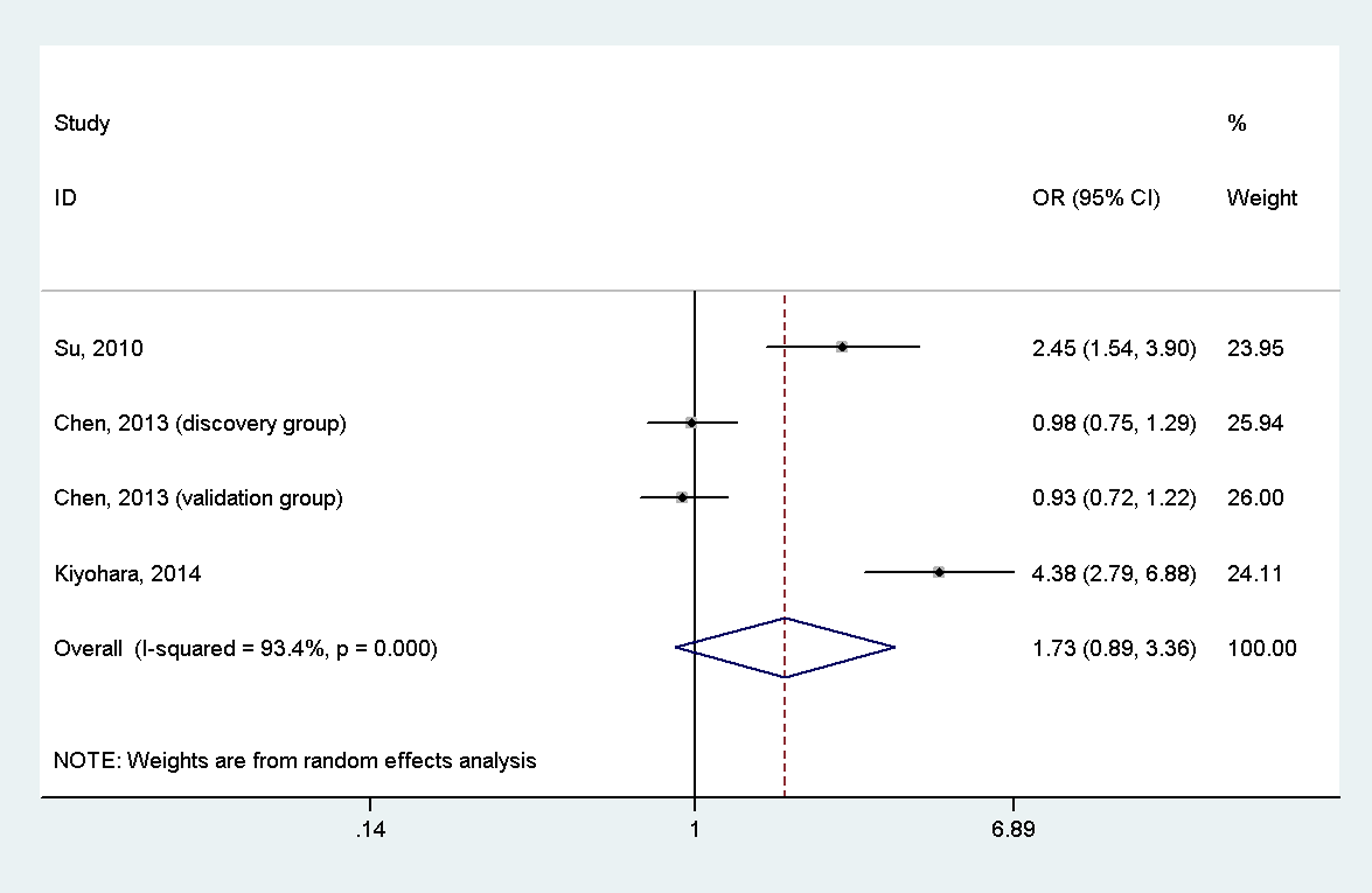


**Figure S2** Forest plot of the association between *IL-6* rs1800796 variant and lung cancer in smokers (dominant model: CC+GC vs. GG)
